# 2-(2-Chloro­pyrimidin-4-yl)-3,5,6,7,8,9-hexahydro-2*H*-1,2,4-triazolo[4,3-*a*]azepin-3-one

**DOI:** 10.1107/S1600536808028900

**Published:** 2008-09-13

**Authors:** Gong-Chun Li, Li-Ye Wang, Zhao-Yang Li, Feng-Ling Yang

**Affiliations:** a College of Chemistry and Chemical Engineering, Xuchang University, Xuchang, Henan Province 461000, People’s Republic of China; b Department of Chemistry, College of Science, Hebei University of Science and Technology, Shijiazhuang 050018, People’s Republic of China

## Abstract

In the title compound, C_11_H_12_ClN_5_O, the triazolone and pyrimidine rings are almost coplanar [dihedral angle = 2.98 (14)°]. The total puckering amplitude *Q_T_* of the seven-membered lactam ring is 0.706 (3) Å.

## Related literature

For the applications of pyrimidine derivatives as pesticides and pharmaceutical agents, see: Condon *et al.* (1993 ▶); as agrochemicals, see: Maeno *et al.* (1990 ▶); as anti­viral agents, see: Gilchrist (1997 ▶); as herbicides, see: Selby *et al.* (2002 ▶). For puckering paramteres, see: Cremer & Pople (1975 ▶).
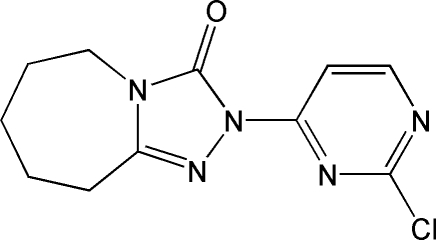


## Experimental

### 

#### Crystal data

C_11_H_12_ClN_5_O
*M* *_r_* = 265.71
Monoclinic, 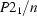

*a* = 8.6810 (16) Å
*b* = 14.718 (3) Å
*c* = 9.4251 (17) Å
β = 92.359 (3)°
*V* = 1203.2 (4) Å^3^
*Z* = 4
Mo *K*α radiation
μ = 0.31 mm^−1^
*T* = 294 (2) K
0.24 × 0.16 × 0.10 mm

#### Data collection

Bruker SMART CCD area-detector diffractometer
Absorption correction: multi-scan (*SADABS*; Sheldrick, 1996 ▶) *T*_min_ = 0.926, *T*_max_ = 0.969
6734 measured reflections
2461 independent reflections
1291 reflections with *I* > 2σ(*I*)
*R*_int_ = 0.047

#### Refinement

*R*[*F* ^2^ > 2σ(*F* ^2^)] = 0.045
*wR*(*F* ^2^) = 0.130
*S* = 1.01
2461 reflections
163 parameters
H-atom parameters constrained
Δρ_max_ = 0.21 e Å^−3^
Δρ_min_ = −0.23 e Å^−3^

### 

Data collection: *SMART* (Bruker, 1999 ▶); cell refinement: *SAINT* (Bruker, 1999 ▶); data reduction: *SAINT*; program(s) used to solve structure: *SHELXS97* (Sheldrick, 2008 ▶); program(s) used to refine structure: *SHELXL97* (Sheldrick, 2008 ▶); molecular graphics: *SHELXTL* (Sheldrick, 2008 ▶); software used to prepare material for publication: *SHELXTL*.

## Supplementary Material

Crystal structure: contains datablocks global, I. DOI: 10.1107/S1600536808028900/at2624sup1.cif

Structure factors: contains datablocks I. DOI: 10.1107/S1600536808028900/at2624Isup2.hkl

Additional supplementary materials:  crystallographic information; 3D view; checkCIF report

## Figures and Tables

**Fig. 1.**
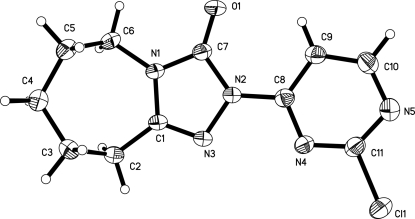
The asymmetric unit of (I), with displacement ellipsoids drawn at the 30% probability level.

## supplementary crystallographic information

### Comment

Pyrimidine derivatives are very important molecules in biology and have many application in the areas of pesticide and pharmaceutical agents (Condon *et al.*, 1993). For example, imazosulfuron, ethirmol and mepanipyrim have been commercialized as agrochemicals (Maeno *et al.*, 1990). Pyrimidine derivatives have also been developed as antiviral agents, such as azidothymidine (AZT), which is the most widely used anti-AIDS drug (Gilchrist, 1997). Recently, a new series of highly active herbicides of substituted azolylpyrimidines were reported (Selby *et al.*, 2002). In order to discover further biologically active pyrimidine compounds, the title compound, (I), was synthesized and its crystal structure determined (Fig. 1).

In the crystal structure of (I), the triazolone and pyrimidine rings are almost coplanar. The dihedral angle between them is 2.99 (18)°. The total puckering amplitude Q_T_ (Cremer & Pople, 1975) of the seven-membered lactam ring gives a quantitative evaluation of puckering being 0.706 (3) Å.

### Experimental

The reaction of 6,7,8,9-tetrahydro-2*H*-[1,2,4]triazolo[4,3-*a*]azepin-3(5*H*)-one (0.184 g, 1.2 mmol) with 4-(3-chlorophenoxy)-2-chloropyrimidine (0.241 g, 1 mmol) in the precence of potassium carbonate (0.207 g, 1.5 mmol) was carried out in N,*N*-dimethylformamide (20 ml) at 343 K overnight. The reaction was cooled and partitioned between 20 ml dichloromethane and 20 ml water. The aqueous layer was extracted with dichloromethane. After removal of the solvent, colourless crystals were obtained by recrystallization from ethyl acetate solution by slow evaporation (yield 30%).

### Refinement

All H atoms were placed in calculated positions, with C—H = 0.93 or 0.97 Å, and refined using a riding model, with *U*_iso_(H) = 1.2U_eq_(C).

### Crystal data

**Table:**

C_11_H_12_ClN_5_O	*F*(000) = 552
*M**_r_* = 265.71	*D*_x_ = 1.467 Mg m^−^^3^
Monoclinic, *P*2_1_/*n*	Mo *K*α radiation, λ = 0.71073 Å
Hall symbol: -P 2yn	Cell parameters from 1409 reflections
*a* = 8.6810 (16) Å	θ = 2.6–21.9°
*b* = 14.718 (3) Å	µ = 0.31 mm^−^^1^
*c* = 9.4251 (17) Å	*T* = 294 K
β = 92.359 (3)°	Prism, colourless
*V* = 1203.2 (4) Å^3^	0.24 × 0.16 × 0.10 mm
*Z* = 4	

### Data collection

**Table:**

Bruker SMART CCD area-detector diffractometer	2461 independent reflections
Radiation source: fine-focus sealed tube	1291 reflections with *I* > 2σ(*I*)
graphite	*R*_int_ = 0.047
φ and oω scans	θ_max_ = 26.4°, θ_min_ = 2.6°
Absorption correction: multi-scan (SADABS; Sheldrick, 1996)	*h* = −10→8
*T*_min_ = 0.926, *T*_max_ = 0.969	*k* = −18→17
6734 measured reflections	*l* = −11→11

### Refinement

**Table:**

Refinement on *F*^2^	Primary atom site location: structure-invariant direct methods
Least-squares matrix: full	Secondary atom site location: difference Fourier map
*R*[*F*^2^ > 2σ(*F*^2^)] = 0.045	Hydrogen site location: inferred from neighbouring sites
*wR*(*F*^2^) = 0.130	H-atom parameters constrained
*S* = 1.01	*w* = 1/[σ^2^(*F*_o_^2^) + (0.0559*P*)^2^ + 0.1252*P*] where *P* = (*F*_o_^2^ + 2*F*_c_^2^)/3
2461 reflections	(Δ/σ)_max_ = 0.002
163 parameters	Δρ_max_ = 0.21 e Å^−^^3^
0 restraints	Δρ_min_ = −0.23 e Å^−^^3^

### Special details

**Table:**

Geometry. All e.s.d.'s (except the e.s.d. in the dihedral angle between two l.s. planes) are estimated using the full covariance matrix. The cell e.s.d.'s are taken into account individually in the estimation of e.s.d.'s in distances, angles and torsion angles; correlations between e.s.d.'s in cell parameters are only used when they are defined by crystal symmetry. An approximate (isotropic) treatment of cell e.s.d.'s is used for estimating e.s.d.'s involving l.s. planes.
Refinement. Refinement of *F*^2^ against ALL reflections. The weighted *R*-factor *wR* and goodness of fit *S* are based on *F*^2^, conventional *R*-factors *R* are based on *F*, with *F* set to zero for negative *F*^2^. The threshold expression of *F*^2^ > σ(*F*^2^) is used only for calculating *R*-factors(gt) *etc*. and is not relevant to the choice of reflections for refinement. *R*-factors based on *F*^2^ are statistically about twice as large as those based on *F*, and *R*- factors based on ALL data will be even larger.

### Fractional atomic coordinates and isotropic or equivalent isotropic displacement parameters (Å^2^)

**Table:**

	*x*	*y*	*z*	*U*_iso_*/*U*_eq_	
Cl1	1.10477 (10)	0.59079 (6)	0.12418 (10)	0.0753 (3)	
O1	0.6140 (2)	0.37929 (13)	0.5659 (2)	0.0643 (6)	
N1	0.7797 (2)	0.25640 (15)	0.5395 (2)	0.0446 (6)	
N2	0.8353 (3)	0.38014 (15)	0.4293 (2)	0.0462 (6)	
N3	0.9482 (3)	0.31650 (15)	0.3968 (2)	0.0505 (6)	
N4	0.9548 (3)	0.48516 (15)	0.2886 (2)	0.0488 (6)	
N5	0.8656 (3)	0.63806 (16)	0.2619 (3)	0.0603 (7)	
C1	0.9097 (3)	0.24464 (18)	0.4640 (3)	0.0463 (7)	
C2	0.9975 (4)	0.1581 (2)	0.4627 (3)	0.0600 (9)	
H2A	1.0848	0.1655	0.4028	0.072*	
H2B	0.9318	0.1111	0.4206	0.072*	
C3	1.0563 (3)	0.1263 (2)	0.6091 (3)	0.0580 (8)	
H3A	1.1356	0.0808	0.5974	0.070*	
H3B	1.1035	0.1775	0.6591	0.070*	
C4	0.9327 (4)	0.0867 (2)	0.6996 (3)	0.0596 (8)	
H4A	0.9824	0.0608	0.7844	0.072*	
H4B	0.8829	0.0373	0.6472	0.072*	
C5	0.8092 (4)	0.1522 (2)	0.7447 (3)	0.0582 (8)	
H5A	0.7464	0.1216	0.8127	0.070*	
H5B	0.8593	0.2029	0.7933	0.070*	
C6	0.7045 (3)	0.18922 (19)	0.6280 (3)	0.0517 (8)	
H6A	0.6676	0.1394	0.5684	0.062*	
H6B	0.6157	0.2171	0.6696	0.062*	
C7	0.7269 (3)	0.34300 (19)	0.5188 (3)	0.0456 (7)	
C8	0.8418 (3)	0.46838 (18)	0.3766 (3)	0.0431 (7)	
C9	0.7356 (3)	0.53480 (18)	0.4117 (3)	0.0492 (7)	
H9	0.6562	0.5230	0.4723	0.059*	
C10	0.7550 (4)	0.6178 (2)	0.3520 (3)	0.0601 (9)	
H10	0.6869	0.6637	0.3752	0.072*	
C11	0.9571 (3)	0.5687 (2)	0.2385 (3)	0.0502 (7)	

### Atomic displacement parameters (Å^2^)

**Table:**

	*U*^11^	*U*^22^	*U*^33^	*U*^12^	*U*^13^	*U*^23^
Cl1	0.0592 (6)	0.0746 (6)	0.0940 (7)	−0.0024 (5)	0.0242 (5)	0.0263 (5)
O1	0.0519 (13)	0.0689 (14)	0.0746 (14)	0.0205 (11)	0.0324 (11)	0.0145 (11)
N1	0.0400 (14)	0.0512 (14)	0.0438 (13)	0.0063 (11)	0.0147 (11)	0.0071 (11)
N2	0.0433 (14)	0.0459 (14)	0.0508 (14)	0.0082 (11)	0.0167 (11)	0.0065 (11)
N3	0.0451 (15)	0.0494 (14)	0.0587 (15)	0.0120 (12)	0.0224 (12)	0.0085 (12)
N4	0.0409 (14)	0.0527 (15)	0.0535 (15)	0.0011 (11)	0.0097 (12)	0.0097 (12)
N5	0.0670 (19)	0.0487 (15)	0.0658 (17)	0.0016 (13)	0.0091 (14)	0.0049 (13)
C1	0.0444 (17)	0.0501 (17)	0.0456 (16)	0.0077 (14)	0.0171 (13)	0.0042 (14)
C2	0.062 (2)	0.0566 (18)	0.064 (2)	0.0175 (16)	0.0283 (16)	0.0089 (16)
C3	0.0465 (19)	0.0531 (18)	0.075 (2)	0.0107 (15)	0.0113 (16)	0.0119 (16)
C4	0.058 (2)	0.0604 (19)	0.0612 (19)	0.0065 (16)	0.0093 (16)	0.0161 (16)
C5	0.060 (2)	0.067 (2)	0.0485 (17)	0.0048 (16)	0.0130 (15)	0.0141 (16)
C6	0.0426 (17)	0.0586 (18)	0.0551 (18)	−0.0039 (15)	0.0166 (14)	0.0098 (15)
C7	0.0424 (17)	0.0532 (17)	0.0418 (16)	0.0049 (14)	0.0102 (13)	0.0054 (14)
C8	0.0407 (16)	0.0493 (17)	0.0393 (16)	−0.0002 (13)	0.0015 (13)	0.0012 (13)
C9	0.0495 (18)	0.0514 (18)	0.0472 (17)	0.0023 (15)	0.0100 (14)	−0.0039 (14)
C10	0.070 (2)	0.0496 (18)	0.061 (2)	0.0104 (17)	0.0104 (18)	−0.0016 (16)
C11	0.0445 (18)	0.0543 (19)	0.0520 (18)	−0.0051 (15)	0.0045 (14)	0.0060 (15)

### Geometric parameters (Å, °)

**Table:**

Cl1—C11	1.739 (3)	C2—H2B	0.9700
O1—C7	1.216 (3)	C3—C4	1.514 (4)
N1—C7	1.366 (3)	C3—H3A	0.9700
N1—C1	1.370 (3)	C3—H3B	0.9700
N1—C6	1.465 (3)	C4—C5	1.516 (4)
N2—C8	1.392 (3)	C4—H4A	0.9700
N2—N3	1.399 (3)	C4—H4B	0.9700
N2—C7	1.400 (3)	C5—C6	1.500 (4)
N3—C1	1.284 (3)	C5—H5A	0.9700
N4—C11	1.318 (3)	C5—H5B	0.9700
N4—C8	1.333 (3)	C6—H6A	0.9700
N5—C11	1.317 (4)	C6—H6B	0.9700
N5—C10	1.341 (4)	C8—C9	1.393 (4)
C1—C2	1.485 (4)	C9—C10	1.358 (4)
C2—C3	1.525 (4)	C9—H9	0.9300
C2—H2A	0.9700	C10—H10	0.9300
			
C7—N1—C1	108.8 (2)	H4A—C4—H4B	107.4
C7—N1—C6	123.8 (2)	C6—C5—C4	116.1 (2)
C1—N1—C6	127.4 (2)	C6—C5—H5A	108.3
C8—N2—N3	120.5 (2)	C4—C5—H5A	108.3
C8—N2—C7	128.1 (2)	C6—C5—H5B	108.3
N3—N2—C7	111.4 (2)	C4—C5—H5B	108.3
C1—N3—N2	104.1 (2)	H5A—C5—H5B	107.4
C11—N4—C8	114.7 (2)	N1—C6—C5	113.1 (2)
C11—N5—C10	112.7 (2)	N1—C6—H6A	109.0
N3—C1—N1	112.9 (2)	C5—C6—H6A	109.0
N3—C1—C2	124.0 (2)	N1—C6—H6B	109.0
N1—C1—C2	123.2 (2)	C5—C6—H6B	109.0
C1—C2—C3	114.1 (2)	H6A—C6—H6B	107.8
C1—C2—H2A	108.7	O1—C7—N1	128.9 (2)
C3—C2—H2A	108.7	O1—C7—N2	128.3 (3)
C1—C2—H2B	108.7	N1—C7—N2	102.8 (2)
C3—C2—H2B	108.7	N4—C8—N2	115.9 (2)
H2A—C2—H2B	107.6	N4—C8—C9	121.9 (3)
C4—C3—C2	114.1 (3)	N2—C8—C9	122.2 (2)
C4—C3—H3A	108.7	C10—C9—C8	116.0 (3)
C2—C3—H3A	108.7	C10—C9—H9	122.0
C4—C3—H3B	108.7	C8—C9—H9	122.0
C2—C3—H3B	108.7	N5—C10—C9	124.5 (3)
H3A—C3—H3B	107.6	N5—C10—H10	117.8
C3—C4—C5	116.0 (3)	C9—C10—H10	117.8
C3—C4—H4A	108.3	N5—C11—N4	130.2 (3)
C5—C4—H4A	108.3	N5—C11—Cl1	115.1 (2)
C3—C4—H4B	108.3	N4—C11—Cl1	114.7 (2)
C5—C4—H4B	108.3		
			
C8—N2—N3—C1	178.5 (2)	C6—N1—C7—N2	179.7 (2)
C7—N2—N3—C1	−0.2 (3)	C8—N2—C7—O1	2.1 (5)
N2—N3—C1—N1	−0.3 (3)	N3—N2—C7—O1	−179.3 (3)
N2—N3—C1—C2	−179.5 (3)	C8—N2—C7—N1	−177.9 (3)
C7—N1—C1—N3	0.8 (3)	N3—N2—C7—N1	0.7 (3)
C6—N1—C1—N3	−179.7 (3)	C11—N4—C8—N2	179.0 (2)
C7—N1—C1—C2	179.9 (3)	C11—N4—C8—C9	0.0 (4)
C6—N1—C1—C2	−0.6 (4)	N3—N2—C8—N4	3.9 (4)
N3—C1—C2—C3	120.7 (3)	C7—N2—C8—N4	−177.6 (2)
N1—C1—C2—C3	−58.3 (4)	N3—N2—C8—C9	−177.1 (2)
C1—C2—C3—C4	75.0 (4)	C7—N2—C8—C9	1.4 (4)
C2—C3—C4—C5	−65.9 (4)	N4—C8—C9—C10	−0.6 (4)
C3—C4—C5—C6	66.4 (4)	N2—C8—C9—C10	−179.6 (3)
C7—N1—C6—C5	−122.9 (3)	C11—N5—C10—C9	−1.2 (5)
C1—N1—C6—C5	57.7 (4)	C8—C9—C10—N5	1.3 (5)
C4—C5—C6—N1	−73.1 (4)	C10—N5—C11—N4	0.4 (5)
C1—N1—C7—O1	179.1 (3)	C10—N5—C11—Cl1	−179.2 (2)
C6—N1—C7—O1	−0.4 (5)	C8—N4—C11—N5	0.1 (5)
C1—N1—C7—N2	−0.8 (3)	C8—N4—C11—Cl1	179.8 (2)
